# Recent progress in surgical treatment of cervical spine myelopathy – A narrative review

**DOI:** 10.1016/j.jcot.2025.103074

**Published:** 2025-05-26

**Authors:** Jun Ouchida, Hiroaki Nakashima, Sadayuki Ito, Naoki Segi, Ippei Yamauchi, Shiro Imagama

**Affiliations:** Department of Orthopaedics, Nagoya University Graduate School of Medicine, Nagoya, Japan

## Abstract

Surgical techniques and technology for cervical spondylotic myelopathy (CSM) have demonstrated remarkable advancement during the past decade. This narrative review examines the evolution of surgical approaches and technological innovations, focusing on both anterior and posterior techniques. Anterior approaches have progressed through the development of endoscopic procedures, novel decompression methods such as vertebral body sliding osteotomy, and advanced implant materials including 3D-printed customized devices. Posterior approaches have evolved with the integration of endoscopic techniques and refined fixation methods, demonstrating reduced tissue trauma and improved outcomes. The integration of surgical assistance technologies, including navigation systems, robotics, and augmented reality, has enhanced surgical precision while potentially reducing procedural risks. While these advances show promising outcomes in terms of surgical precision and patient recovery, challenges persist regarding technology implementation and cost-effectiveness. Emerging technologies such as artificial intelligence for surgical planning, patient-specific implants, and adjunctive biological therapies may further improve CSM treatment. The optimal application of these innovations requires continued research and careful evaluation to establish their long-term efficacy and safety in clinical practice.

## 1 Introduction

Cervical Spondylotic Myelopathy (CSM), also referred to as Degenerative Cervical Myelopathy (DCM), is a progressive condition involving compression of the cervical spinal cord. This disorder results from age-associated degenerative changes in the cervical spine, such as disc degeneration, osteophyte formation, and ligamentum flavum hypertrophy^1^ This mechanical compression leads to direct neural tissue damage and vascular impairment, resulting in neurological dysfunction. The pathophysiological cascade involves both static factors causing sustained compression and dynamic factors associated with repetitive spinal cord injury during cervical movements. Given the lack of established conservative treatment options, surgical decompression, either with or without fusion, remains the gold standard for managing CSM, with procedures tailored to the specific pathology.^2^

The history of CSM surgery dates back to the mid-20th century, with the development of fundamental anterior and posterior approaches. Contemporary surgical techniques were established through modifications of pioneering procedures, including the anterior cervical discectomy and fusion (ACDF) first described by Smith-Robinson, and Cloward methods, as well as posterior approaches such as laminectomy and laminoplasty.^1^^,^^3^^,^^4^ While these conventional methods have proven effective, they carry risks of tissue damage and potential postoperative complications.^5^ Recent trends in spine surgery emphasize reducing invasiveness while maximizing effectiveness through surgical innovations and technological integration, and CSM procedures must align with these requirements. Furthermore, there remain numerous unresolved issues requiring further investigation, such as the varying outcomes and complication rates associated with different surgical approaches, including anterior versus posterior approaches, the role of fusion, and multi-level procedures.^6^^,^^7^ Amid these challenges, each surgical technique continues to evolve progressively.

In recent years, a transition has occurred from traditional open procedures to minimally invasive surgery (MIS), which employs more advanced techniques. This development reflects the fusion of established surgical expertise with latest technology, leading to more effective and less invasive treatment options for patients with CSM. The primary focus of this review is on recent surgical advancements across different approaches, mainly drawing from literature published during the latter part of the past decade.

The objectives of this narrative review are threefold: first, to analyze recent innovations in both anterior and posterior surgical techniques for cervical myelopathy; second, to describe the impact of surgical adjunct technologies on operative outcomes; and finally, to discuss emerging technologies and their potential applications in cervical myelopathy surgery.

## 2 Advances in anterior surgical approaches

The anterior approach is generally indicated for CSM cases where disc protrusion is limited to short segments or when the primary pathology is situated anteriorly. The anterior approach for CSM has undergone significant developments in surgical techniques and materials in recent years, with innovations focusing on minimizing surgical invasion while maximizing effectiveness. Recent advances can be primarily categorized into three areas: MIS techniques including endoscopic surgery, novel methods for decompressing ossified lesions, and implant innovations.

### 2.1 Minimally invasive anterior techniques

Anterior cervical discectomy and fusion (ACDF) involves direct decompression of the spinal canal through the removal of anteriorly protruding discs and osteophytes. ACDF continues to be a commonly performed and well-established treatment for CSM,^8^ Potential complications, including pseudarthrosis, the development of adjacent segment degeneration, and the loss of mobility at the fused levels, remain concerns for surgeons. Endoscopic spine surgery, which initially developed in the lumbar region, has recently been adapted for cervical applications with several reported benefits. The typical advantages of endoscopic spine procedures are smaller skin incisions, less muscle and soft tissue damage, reduced blood loss, and shorter hospital stays after surgery.

・ Endoscopic Anterior Cervical Approach

**Fig. 1:**
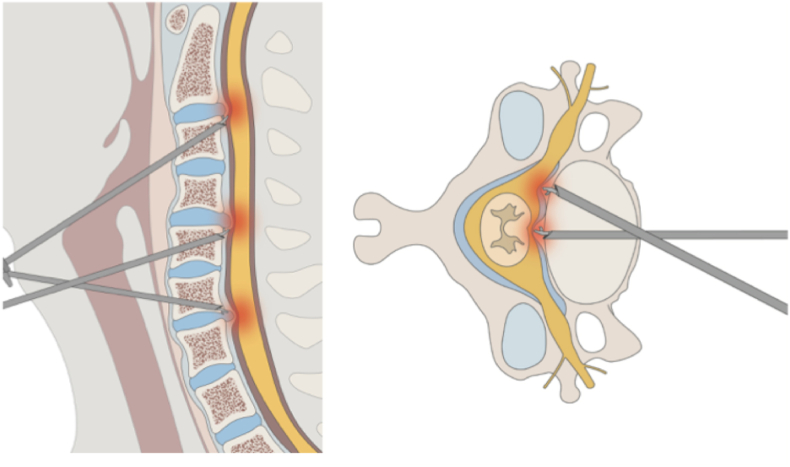
Anterior transcorporeal approach The sagittal (left) and axial (right) views demonstrate the endoscopic trajectory. Various angles of approach through the vertebral body enable wide-ranging neural decompression.

The anterior endoscopic cervical transcorporeal approach is a surgical method used to treat spinal cord and nerve root compression caused by anterior pathologies in short segments with moderate degeneration.^9^ This procedure achieves effective neural decompression by creating an access pathway through the vertebral body without requiring interbody fusion (Fig. 1). In a case series involving 28 CSM patients, the mean operative time was 118 min, and the average hospital stay was 3.5days.^10^ This approach provides an effective surgical option for spinal cord compression pathologies with minimal deformity or degeneration, as it maintains cervical mobility by avoiding the need for disc removal. The procedure, which demands high technical expertise, has been made possible through ongoing improvements in endoscopic instruments and surgical tools, along with three-dimensional anatomical assessment of lesions utilizing CT and MRI imaging technologies.^11^^,^^12^

- Endoscopic-Assisted ACDF: Anterior cervical endoscopic procedures decrease the risk of postoperative dysphagia, hoarseness, and bleeding by minimizing the need for soft tissue retraction.^13^ In addition to its minimal invasiveness, this approach provides efficient decompression through superior visualization of posterior endplates and the spinal canal, enhanced by irrigation and angled endoscopes.^14^ However, conventional open microscopic techniques remain the predominant method for anterior cervical fusion, with limited research demonstrating the superiority of endoscopic assistance.^15^

### 2.2 Vertebral body sliding osteotomy

**Fig. 2:**
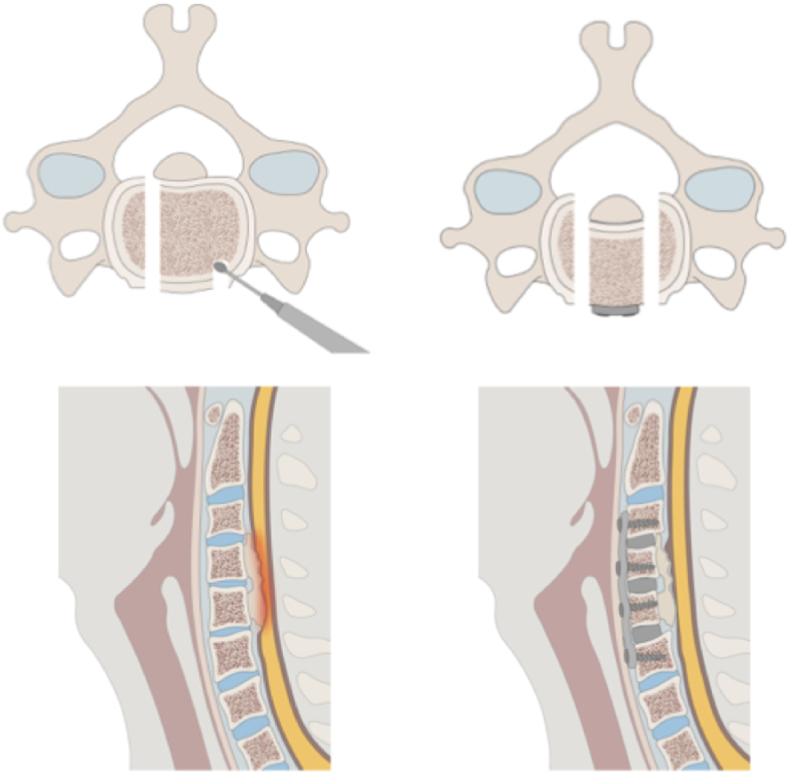
Vertebral body sliding osteotomy (Upper and lower left)) Anterior neural compression caused by ossification. The vertebral body is drilled in parallel at the necessary width using a high-speed drill. (Upper and lower right) After cage insertion between vertebral bodies, the anterior surface of the target vertebral body is resected, and screws are placed through a plate spanning the cranial and caudal ends to pull the vertebrae together, achieving neural decompression.

CSM frequently presents with rigid kyphotic alignment, a condition where posterior decompression alone may not achieve adequate posterior cord migration, potentially resulting in suboptimal neurological improvement. In such cases, multilevel anterior cervical fusion has traditionally been performed to achieve direct dural decompression and restore cervical lordosis. Vertebral body sliding osteotomy (VBSO) is an innovative anterior cervical technique that aims to reduce the risks of dural injury and graft instability linked to traditional methods for treating OPLL.^16^^,^^17^ This technique includes creating a longitudinal osteotomy at the decompression width of the vertebral body and then pulling the bone fragment toward an anteriorly positioned plate using screws, which achieves decompression by displacing the posterior ligamentous and ossified components away from the spinal cord (Fig. 2). A comparative study of 24 VBSO cases and 38 conventional anterior cervical corpectomy and fusion (ACCF) cases showed that VBSO resulted in shorter operative times and less blood loss, while maintaining similar neurological outcomes.^18^ In this smaller cohort study, the VBSO group also showed lower rates of cerebrospinal fluid leakage and pseudarthrosis at 12 months. This approach raises fewer concerns about pseudarthrosis and graft subsidence, and offers potential benefits for patients experiencing spinal cord compression due to both disc space and posterior vertebral body pathologies.^19^

### 2.3 Implant innovations for anterior approach

The evolution of anterior implants has been marked by several significant developments:

- Advanced Cage Materials: ACDF has seen the introduction of porous titanium cages with optimized surface topology for enhanced osseointegration. These new designs incorporate varying pore sizes and interconnectivity patterns based on computational modeling of bone ingrowth patterns. The fabrication of these cage structures has been enabled through advances in additive manufacturing technologies, particularly 3D printing, creating synergistic benefits with titanium's inherent advantages for interbody fusion.^20^ In a follow-up study of 49 patients with porous titanium cages, earlier fusion rates were achieved compared to the PEEK (polyetheretherketone) group (89 % vs 72 % at 6 months postoperatively).^21^ This investigation was limited to a relatively short-term follow-up of one year and excluded cases with concurrent plate fixation. Additionally, a case series involving 76 patients (120 levels) who used 3D printed titanium cages for ACDF showed patient-reported outcomes and fusion rates similar to those of allografts, while also demonstrating less cage subsidence and better maintenance of cervical lordosis.^22^
- Vertebral Body Replacement Cages: In anterior decompression of multilevel lesions, vertebral body replacement cages are utilized. Conventional titanium cages have been associated with postoperative cage subsidence. The mechanisms behind cage subsidence are believed to be linked to the mismatch in material stiffness between titanium mesh and host bone, and the decreased contact area with endplates due to sharp edges following titanium mesh trimming, leading to localized high-stress failure. To overcome these challenges, researchers have investigated biomimetic nonmetallic cages that combine a bone-like material with nylon, which have a stiffness similar to vertebral bone, as well as 3D-printed vertebral bodies (3D-VBs) made from biocompatible titanium alloy powder that mimic patient-specific anatomical structures.^23^ These cages demonstrated lower subsidence rates compared with titanium cages.^24^ Although these novel cages demonstrate favorable clinical outcomes, their cost implications relative to conventional cages require further evaluation of their economic viability.
- Zero-Profile Devices for Multilevel Fixation: Anterior cervical plate-cage fixation (ACPC) is developed to be one of the standard surgical treatments for CSM with cervical deformity and multilevel involvement. However, the addition of anterior cervical approached carries risks of postoperative dysphagia, plate-screw-related complications, and adjacent segment degeneration. Zero-Profile (ZP) devices are characterized by minimal implant protrusion beyond the anterior vertebral surface and may provide superior fixation compared to conventional standalone devices. A follow-up study of 58 cases involving three-level ACDF with ZP implants showed satisfactory long-term clinical and radiological outcomes that were similar to those achieved with anterior cervical plating.^25^ In comparative study of 56 conventional plate fixation cases versus 94 ZP device cases, while conventional plating showed advantages in correcting surgical site curvature, ZP devices demonstrated lower rates of postoperative dysphagia.^26^ These smaller cohort studies revealed no difference in overall complication rates between the groups, highlighting the necessity for larger-scale investigations. ZP devices, which provide additional vertebral anchoring through screws or blades and include fixation mechanisms, have been linked to reports of middle cervical vertebra collapse and vertebral fractures, requiring meticulous intraoperative handling and postoperative radiological monitoring.^27^^,^^28^

### 2.4 Artificial disc replacement for CSM

A systematic review of artificial disc replacement (ADR) for CSM showed that the ADR achieved preferable functional outcomes, as reflected in quality of life (QOL) scores, compared to the ACDF.^29^ ADR offers the advantage of preserving range of motion at the surgical disc level without increasing complications. However, current studies on ADR for CSM are constrained by relatively small sample sizes and short follow-up periods.^30^ To validate the outcomes of ADR, it will be essential to conduct studies with sufficiently large sample sizes and longer follow-up.

## 3 Advances in posterior surgical approaches

Posterior approaches for CSM treatment continue to evolve, with the adaptation of endoscopic techniques bringing innovations focused on tissue preservation and improved surgical invasiveness and patient outcomes. These advancements have resulted in enhanced surgical results while reducing approach-related complications.

### 3.1 Minimally invasive posterior techniques

Recent innovations in minimally invasive surgery (MIS) have transformed traditional posterior approaches: Endoscopic MIS techniques, initially developed for lumbar decompression, have recently been adapted for cervical applications through hardware improvements. In posterior cervical surgery, endoscopic techniques are now being incorporated with laminoplasty and laminectomy procedures.

**Fig. 3:**
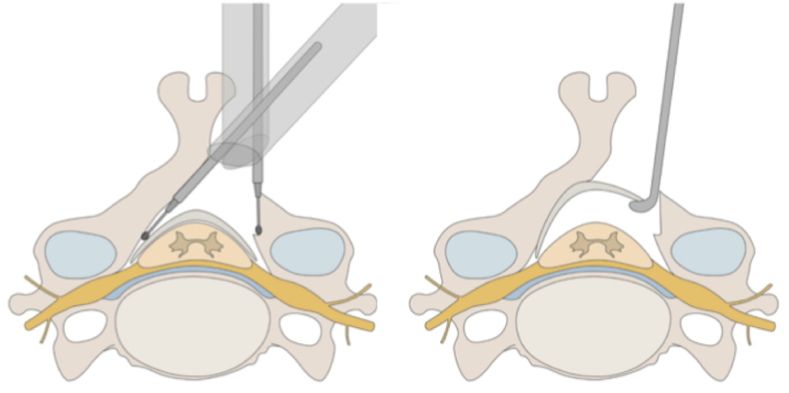
Endoscopic laminotomy (Left) Endoscopic approach from one side to partially resect the spinous process and contralateral lamina while preserving the ligamentum flavum. (Right) After bony decompression, the ligamentum flavum is removed to complete neural decompression.
- Microendoscopic Decompression: The use of high-definition endoscopic equipment combined with specialized tools allows for accurate posterior decompression via minimally invasive approaches. This method significantly reduces injury to posterior soft tissues, limits intraoperative blood loss, shortens the duration of postoperative hospitalization, and promotes more rapid patient recovery.^33^^,^^34^ For patients with pathology confined to a few segments and without marked cervical instability or deformity, endoscopic posterior decompression is considered an effective treatment option. Comparative research has indicated that endoscopic cervical procedures, when evaluated against ACDF, are associated with reduced blood loss and briefer hospital admissions, and are frequently performed using local anesthesia.^35^ In a series reported by Sharma et al., over 220 patients who underwent unilateral endoscopic cervical canal decompression experienced acceptable operative durations, very low average blood loss (mean 32 mL, range 15–90 mL), and brief hospital stays (mean 3.2 days, range 2–5 days).^36^

- Endoscopic Laminotomy: Posterior cervical surgical procedures often result in injury to the paraspinal muscles and ligamentous structures, which are considered key factors in the development of postoperative axial neck pain and reduction of cervical lordosis. Endoscopic-assisted methods, particularly those utilizing a unilateral laminotomy to achieve bilateral decompression, are designed to limit disruption to these posterior elements while still providing effective segmental spinal canal decompression (Fig. 3).^31^ In a long-term follow-up involving patients who underwent microendoscopic laminotomy, neurological improvement was found to be on par with that observed after conventional laminoplasty. However, those treated with the endoscopic technique reported notably lower axial pain scores five years after surgery (VAS 20.8 ± 15.5 compared to 44.3 ± 25.0, p < 0.001), as well as a significant enhancement in cervical lordosis relative to preoperative values (2.9 ± 8.6 versus −2.3 ± 7.1, p = 0.02)^32^

### 3.2 Posterior fixation surgery

Posterior cervical fixation techniques remain a cornerstone in the management of myelopathy associated with instability or dynamic factors contributing to spinal cord compression. While these techniques have not undergone radical transformation recently, significant progress has occurred in navigation systems and specialized devices that enhance surgical precision, particularly for pedicle screw placement.

- Spinal Fixation for OPLL: OPLL is a condition characterized by abnormal ligament ossification, leading to narrowing of the spinal canal and subsequent spinal cord compression in hyperostotic states. Although surgical intervention remains the definitive therapy for OPLL, the necessity and effectiveness of direct decompression are still debated. In a clinical series involving 52 patients treated with spinal fixation alone—without removal of bone or soft tissue—51 individuals experienced notable and immediate postoperative improvement. Furthermore, among 14 patients who were unable to walk and required a wheelchair prior to surgery, 12 regained the ability to walk independently within six months following the procedure.^37^ This evidence suggests that spinal fixation without decompression may offer significant functional benefits for selected OPLL patients.

The decision to perform fusion in cervical spine surgery remains a topic of debate due to the dynamic nature of the cervical spine. This ongoing debate centers on three critical aspects: postoperative spinal mobility, progression of ossification, and the role of spinal alignment in fusion indication.^38^, ^39^, ^40^ These factors require careful individual assessment to determine the optimal surgical approach.

## 4 Advances in surgical assistance technologies

Recent years have witnessed unprecedented integration of advanced technologies in CSM surgery, revolutionizing surgical precision, planning, and execution. These technological developments have considerably enhanced surgical outcomes while reducing procedural risks.

### 4.1 Technological assistance

Surgical assistance technologies have significantly enhanced the precision of anterior procedures:

- Navigation-Assisted Cervical decompression: Navigation systems in anterior cervical surgery enhance the accuracy of decompression, particularly in cases involving complex deformities or challenging ossified lesions^41^ For these anterior approaches, the reference frame is typically affixed to the cranial stabilization device, allowing for real-time three-dimensional imaging via intraoperative CT.^42^
- Augmented Reality Guidance: Augmented Reality (AR) technology in anterior cervical surgery enhances safety and precision by overlaying virtual 3D anatomical models onto the surgeon's real-time view of the operative field. These models, generated from preoperative CT and MRI scans, allow surgeons to visually identify and confirm the locations of critical structures such as vertebral arteries, nerve roots, and the spinal cord during the procedure.^43^ AR is particularly promising for complex surgeries that demand meticulous manipulation of intricate anatomy, such as the floating method for decompression in cases of OPLL.^44^ Clinical reports have demonstrated that AR-assisted microscopic visualization improves intraoperative orientation, facilitates accurate identification of ossified lesions, and helps ensure sufficient decompression without increasing complication rates.

### 4.2 Robotic surgical assistance

The complexity of cervical pedicle screw placement significantly differs from lumbar and thoracic procedures due to anatomical challenges, pedicle angulation, narrow spinal canal, and cervical mobility. Robotic-assisted (RA) surgery provides advantages in precise instrument placement independent of surgeon experience and skill, while reducing radiation exposure for medical staff. Robotic technology in CSM surgery has progressed from experimental applications to practical clinical use.^45^^,^^46^ The use of RA screw placement resulted in a significant decrease in complication rates, intraoperative blood loss, hospital stay, and radiation exposure when compared to conventional free hand technique method.^47^, ^48^, ^49^

While robotic assistance appears to contribute to improved accuracy of screw placement compared to freehand methods, concerns remain regarding increased operative time.^50^^,^^51^

### 4.3 Technological integration in posterior surgery

Advanced technologies have significantly enhanced the precision and safety of posterior procedures:

- 3D-Printed Patient-Specific Guides: The development of customized surgical guides based on preoperative imaging data enables accurate cervical screw placement while reducing intraoperative radiation exposure and operative time.^52^^,^^53^ 3D printing technology allows for the creation of patient-specific drill guides that closely match individualized virtual vertebral models. Clinical studies have demonstrated that these guides enable accurate placement of pedicle and lateral mass screws, with postoperative assessments confirming that the actual screw trajectories closely align with preoperative surgical plans. Analysis of screw insertion using these custom guides reported safe screw insertion and no reported cases of neurovascular injury, facet joint violation, or pedicle wall breach.^54^

- Advanced Neuromonitoring: Multimodal intraoperative monitoring (IOM) serves as a critical diagnostic tool for assessing spinal cord integrity during spinal surgery. It commonly utilizes motor evoked potentials (MEP) and somatosensory evoked potentials (SEP) to monitor neural function. In a study involving patients with cervical spondylotic myelopathy (CSM), improvements in MEP during IOM were linked to better neurological outcomes one month after surgery.^55^

The routine use of IOM for ACDF remains controversial. A retrospective review of 15,395 ACDF patients^5^ revealed IOM usage in 2627 cases (17.1 %). Despite the variety of modalities employed, the study indicated that IOM did not further prevent postoperative neurological complications compared to cases without IOM.

## 5 Discussion

### 5.1 Decision of surgical approach

The comparative effectiveness of anterior and posterior surgical approaches in cervical spondylotic myelopathy (CSM) continues to be a focus of clinical investigation. Evidence from a large database study of 1141 CSM patients with severe neck pain undergoing three- or four-level procedures indicates that both anterior cervical discectomy and fusion (ACDF) and posterior cervical laminectomy and fusion (PCLF) result in similar improvements in postoperative neck pain at 3, 12, and 24 months^7^ However, multivariable analyses show that multilevel ACDF is associated with superior outcomes at 24 months regarding functional status, quality of life, and likelihood of returning to preoperative activity levels compared to PCLF.

In a comprehensive analysis involving over 33,000 patients with cervical myelopathy, significant baseline differences were observed between those undergoing anterior and posterior surgeries, including variations in age, comorbidities, myelopathy severity, unemployment rates, and hospital stay lengths. After adjusting for these disparities, patients who received anterior surgery were more likely to achieve substantial improvements in the Neck Disability Index (NDI) during both short-term and long-term follow-up periods.^56^

In actual clinical practice, surgical decision-making incorporates multiple factors beyond anatomical characteristics, including patients' physical and social background, comorbidities, surgeon expertise, and access to medical resources.^7^^,^^57^ In the elderly population, it should be noted that patients with mild CSM may not achieve sufficient surgical benefit compared to the natural course of the disease.^58^

### 5.2 Endoscopic surgery for CSM

Endoscopic techniques in cervical spine surgery offer several advantages, including shorter hospital stays and reduced intraoperative blood loss.^59^ These methods can be applied to both anterior and posterior approaches and have a broad range of indications. For instance, endoscopic posterior decompression is particularly suitable for cases where compression occurs slightly lateral to the center (paracentral).^60^

These surgical approaches and techniques require comprehensive understanding of cervical spine anatomy and pathological mechanisms. The successful execution of MIS procedures depends on the capabilities of endoscopic systems, and managing complications may require advanced technical expertise. Anterior endoscopic cervical surgery often accompanied by several common complications, such as dysphagia, hematoma, endplate injury, and voice disturbance.^61^ In contrast, complications reported with the posterior approach encompass transient sensory disturbances, recurrent neurological deficits, dural tears, upper extremity motor dysfunction, and persistent arm pain. Surgeons must be aware of their institutional equipment limitations regarding surgical indications and be prepared to manage potential complications.^11^^,^^36^

### 5.3 Impact of technological integration

Navigation systems, robotics, and advanced imaging have significantly improved surgical precision, particularly in complex cases. However, the learning curve associated with these technologies and their cost implications remain significant considerations for widespread adoption.^62^ The integration of these technologies requires substantial infrastructure investment and specialized training, which may limit their accessibility in some clinical settings.

### 5.4 Novel technologies and Therapeutic approaches in CSM surgery

Several emerging trends are shaping the future of CSM surgery. First is the integration of artificial intelligence with CSM treatment. Recent studies have demonstrated that machine learning approaches using MRI images can predict and diagnose CSM, as well as estimate prognosis.^63^^,^^64^ The development of machine learning algorithms may identify specific patient populations most likely to benefit from surgery, thereby informing surgical decision-making and outcome prediction.^65^

Next is the advancement of personalized medicine through 3D printing technology. From custom implants to individualized surgical planning, patient-specific surgical solutions show potential for improving outcomes. Reports in this field include customized cervical locking plates for complex anatomical cases and patient-specific vertebral body replacement implants.^66^^,^^67^

**Finally, smart implants integrated with sensor technology provide real-time monitoring capabilities for spinal cord compression and local loads at implantation sites postoperatively**.^68^ These devices enable real-time assessment of healing and early detection of potential complications. However, clinical implementation requires thorough evaluation of sensor durability, data interpretation protocols, and cost-effectiveness.

## 6 Conclusion

The past decade has demonstrated consistent progress in surgical techniques and technology for CSM. Surgical precision and safety have improved through the integration of navigation systems, robotics, and advanced materials, while minimally invasive approaches have led to reduced surgical complications. Despite ongoing challenges in technology implementation and cost-effectiveness, continued innovation suggests further improvements in surgical outcomes. The evolution of artificial intelligence, personalized medicine, and biological therapies promises to further transform CSM surgery, enhancing patient care. Successful clinical implementation of these emerging technologies depends on continued research and rigorous evaluation.

## CRediT authorship contribution statement

**Jun Ouchida:** Writing – original draft, Conceptualization. **Hiroaki Nakashima:** Writing – review & editing, Supervision. **Sadayuki Ito:** Supervision. **Naoki Segi:** Supervision. **Ippei Yamauchi:** Supervision, Yukihito Ode, Supervision. **Shiro Imagama:** Supervision.

## Guardian/patient's consent

Not applicable as this is a review article and does not involve patient data or interventions.

## Ethical statement

Not applicable as this is a review article and does not involve human subjects research, patient data collection, or interventions.

## Funding statement

No funds were received in support of this work. No benefits in any form have been or will be received from a commercial party related directly or indirectly to the subject of this manuscript.

## Declaration of competing interest

The authors declare that they have no known competing financial interests or personal relationships that could have appeared to influence the work reported in this paper.
